# Mangosteen Pericarp Extract Supplementation Boosts Antioxidant Status via Rebuilding Gut Microbiota to Attenuate Motor Deficit in 6-OHDA-Induced Parkinson’s Disease

**DOI:** 10.3390/antiox11122396

**Published:** 2022-12-02

**Authors:** Bira Arumndari Nurrahma, Tu-Hsueh Yeh, Rong-Hong Hsieh, Shu-Ping Tsao, Chia-Wen Chen, Yen-Peng Lee, Chun-Hsu Pan, Hui-Yu Huang

**Affiliations:** 1 Graduate Institute of Metabolism and Obesity Sciences, College of Nutrition, Taipei Medical University, Taipei 11031, Taiwan; 2 Department of Neurology, Taipei Medical University Hospital, Taipei 11031, Taiwan; 3 School of Medicine, Taipei Medical University, Taipei 11031, Taiwan; 4 School of Nutrition and Health Sciences, College of Nutrition, Taipei Medical University, Taipei 11031, Taiwan; 5 Ph.D. Program in Drug Discovery and Development Industry, College of Pharmacy, Taipei Medical University, Taipei 11031, Taiwan; 6 School of Pharmacy, Taipei Medical University, Taipei 11031, Taiwan; 7 Neuroscience Research Center, Taipei Medical University, Taipei 11031, Taiwan; 8 Research Center for Digestive Medicine, Taipei Medical University Hospital, Taipei 11031, Taiwan

**Keywords:** mangosteen pericarp, Parkinson’s disease, antioxidant, fecal microbiota composition, 6-hydroxydopamine

## Abstract

Oxidative stress and gut dysbiosis have been known to precede Parkinson’s disease (PD). An antioxidant-rich product, mangosteen pericarp (MP), has the ability to counterbalance excessive free radicals and the imbalanced gut microbiota composition, suggesting the MP’s capacity to delay PD progression. In this study, we explored the effects of two doses of MP extract in a unilateral 6-hydroxydopamine (6-OHDA)-induced PD rat model. We revealed that the 8-week supplementation of a low dose (LMP) and a high dose of the MP extract (HMP) improved motor function, as observed in decreased contralateral rotation, improved time spent on rod, and higher dopamine binding transporter (DAT) in the substantia nigra pars compacta (SNc). The MP extract, especially the HMP, also increased antioxidant-related gene expressions, restored muscle mitochondrial function, and remodeled fecal microbiota composition, which were followed by reduced reactive oxygen species levels in brain and inflammation in plasma. Importantly, bacterial genera *Sutterella*, *Rothia*, and *Aggregatibacter*, which were negatively correlated with antioxidant gene expressions, decreased in the HMP group. It is imperative to note that in addition to directly acting as an antioxidant to reduce excessive free radicals, MP extract might also increase antioxidant state by rebuilding gut microbiota, thereby enhanced anti-inflammatory capacity and restored mitochondrial function to attenuate motor deficit in 6-OHDA-induced PD-like condition. All in all, MP extract is a potential candidate for auxiliary therapy for PD.

## 1. Introduction

Parkinson’s disease (PD) is a progressive neurodegenerative disease that affected over 8 million individuals globally in 2019 [1]. The development of PD damages dopaminergic neurons in the basal ganglia circuit, particularly in the substantia nigra pars compacta (SNc), which consequently results in deprivation of the neurotransmitter dopamine and affects normal motor function, leading to motor function impairments, for instance bradykinesia, rigidity, tremors, and gait dysfunction [2]. In addition to motor dysfunctions, various non-motor dysfunctions (e.g., gut dysbiosis, constipation, sleep disorders, depression, and dysphagia) have been reported to follow the loss of dopaminergic neurons in PD [2,3]. The exact pathogenesis of PD remains obscure. However, several lines of evidence suggest that the progression of oxidative stress [4,5], inflammation [6,7], and mitochondrial dysfunction [8,9,10] in the brain—along with gut microbiota dysbiosis [3,11]—may contribute to the development of PD.

The correlation between the progression PD and oxidative stress has been discussed extensively. Oxidative stress occurs when there is an imbalance between the levels of free radicals, such as reactive oxygen species (ROS), and antioxidants. Mitochondrial oxidative phosphorylation is one of the major contributors to the generation of ROS products. ROS products such as the superoxide anion and hydrogen peroxide are generated by the electron transport chain processes in cellular energy production involving oxidative phosphorylation [12]. The generation of these ROS products is exacerbated in PD [13,14] due to depletion of mitochondrial proteins (i.e., complexes I, II, and IV) [8,9,10] and the presence of mitochondrial dysfunction [15]. Antioxidants such as superoxide dismutase (SOD) and glutathione peroxidase (GPx) are innately produced to neutralize the ROS. However, in the pathological state of PD, the host fails to produce sufficient levels of antioxidants to counterbalance ROS [16,17]. As a result, accumulation of ROS further damages mitochondria and aggravates dopaminergic neuron loss [12,18]. Moreover, oxidative stress can induce proinflammatory responses in the brain by activating microglia, which promote neuronal degeneration [7]. Additionally, impaired mitochondrial function has not only been reported in the brain of patients with PD, but also in the peripheral tissues, including the skeletal muscles [19,20].

In the past few years, gut dysbiosis has been suggested as a factor involved in the development of PD [3]. Increased abundances of pathogenic bacteria and decreased abundances of commensal bacteria were documented in several clinical studies of patients with PD [11,21,22,23,24,25]. An imbalance in the gut microbiota composition towards proinflammatory bacteria leads to increased inflammatory responses within the host [26]. The antioxidant capacity within the host may also be reduced by decreases in the abundances of short-chain fatty acid-producing bacteria [27,28]. Additionally, the bidirectional axis between the gut and the brain may allow pathogenic bacteria in the gut to promote neuronal degeneration in the brain in PD [29]. Thus, it is imperative to explore PD-related gut dysbiosis since the gastrointestinal disorders in patients with PD occur years before the development of motor deficits [30]. Moreover, this evidence further suggests that the progression of PD is induced by various factors and that the related molecular impairments are not limited to the brain. Thus, targeting the PD-related pathologies that occur in several organs may offer better neuroprotective effects and may even avert the progression of this disease.

Mangosteen (*Garcinia mangostana* Linn.) is a fruit rich in polyphenols [31]. Its pericarp contains various bioactive compounds including xanthones (e.g., α-mangostin, β-mangostin, and γ-mangostin), anthocyanins, and proanthocyanidins [32,33]. Furthermore, xanthones, benzophenones, and anthocyanins within mangosteen pericarp (MP) extract have been reported to possess antioxidant [34], anti-inflammatory [35], antiobesity, antiulcer, and anticancer properties [32] and exert antimicrobial action against pathogenic bacteria [36]. One study [34] reported that the neuroprotective effects of α-mangostin, a phytochemical found in MP, improved motor function and memory function in rats with rotenone-induced PD by decreasing oxidative stress (i.e., reduced nitrite and malondialdehyde (MDA) and increased glutathione (GSH)). In addition, α-mangostin can reduce neuroinflammation [35] and protect neuronal cells from hydrogen peroxide-induced injury [37].

Polyphenols are abundant in MP and can affect the health of the host through modulating the gut microbiota. Polyphenols and the gut microbiota can exert synergistic health benefits to the host as polyphenols can modify the gut microbiota composition and metabolite by selectively affecting bacterial cell membranes and the microbiota can improve the bioavailability of polyphenols by transforming them into bioavailable metabolites [38]. Moreover, MP extract has been reported to inhibit the growth of a pathogenic bacterial species [39], suggesting MP has the capacity to alter the gut microbiota.

Current therapies for PD, such as L-DOPA drugs, only alleviate the motor symptoms and do not target the pathological mechanisms, such as oxidative stress and gut dysbiosis. Therefore, novel therapies that target disease-specific pathologies urgently need to be identified. The bioactive compounds in MP exhibited antioxidant and anti-inflammatory activities and altered the gut microbiota composition in various studies, indicating MP has potential as an auxiliary therapy for the treatment of PD. Therefore, in this study, we investigated the therapeutic effects of an MP extract on striatal and SNc dopamine transporter (DAT) binding activity and motor function in a rat model of 6-OHDA-induced PD-like motor deficits. Furthermore, we explored the potential effects of MP supplementation on regulatory pathways in a PD-like condition, particularly antioxidant defense mechanisms and the function of mitochondria in both the brain and muscles, along with inflammatory markers in blood plasma and microbiota profiles in fecal samples. To the best of our knowledge, this is the first study to link the effects of MP on antioxidant defense mechanisms in the brain and muscles with fecal microbiota profiles in a PD-like condition. Moreover, this work provides new insight on the potential of improving the antioxidant capacities and mitochondrial function of muscles to delay the progressive motor deficits in rats with 6-OHDA-induced PD.

## 2. Materials and Methods

### 2.1. Extraction and Preparation of MP

Mangosteen pericarp (MP) extract was a gift of Prof. Rong-Hong Hsieh (School of Nutrition and Health Sciences, College of Nutrition, Taipei Medical University, Taipei City, Taiwan). Briefly, pericarp of *Garcinia mangostana* L. was dried, ground, extracted in ethanol (pericarp powder:ethanol = 1:10, *w*/*v*) for one day at room temperature, and filtered to obtain mangosteen pericarp ethanol extract solution. The ethanol extract solution was vacuum concentrated. Different percentages of aqueous ethanol solution were added at low temperature to purify the extract. Finally, the extract was dried using an air-spray dryer and ground using a grinder.

The MP extract was assessed by analytic high-performance liquid chromatography (HPLC) using a 250 × 4.6 mm i.d., 5 µm Waters Spherisorb ODS-2 column (Milford, MA, USA). The mobile phase was: solvent A, 0.1% formic acid solution; and solvent B, acetonitrile. Elution conditions were 0–25 min of 35–0% A to B (linear gradient); 25–28 min of 0–35% A to B (linear gradient); 28–30 min of 35–35% A to B at a flow rate of 1.0 mL/min using a Jasco MD-910 photo diode array detector (Tokyo, Japan) at 255 nm wavelength. A standard calibration curve of α-mangostin and γ-mangostin was obtained for a series of standard compound concentrations, then quantification of the index compound in the extract was performed by HPLC analysis. The peak area of the candidate compound was defined in the chromatogram of the extract and its content was calculated from the standard calibration curve. Each gram of MP extract powder contains 470 mg of α-mangostin and 80 mg of γ-mangostin (Figure S1).

For supplementation purposes, the MP extract powder was dissolved in 0.5% carboxymethyl cellulose and sonicated for 1 h. Fresh stock solutions of low- (LMP) and high-concentration MP (HMP) were prepared every day before administration to the rats. The normal control (NC) and PD groups received 0.5% carboxymethyl cellulose.

### 2.2. Animals and Study Design

**Figure 1:**
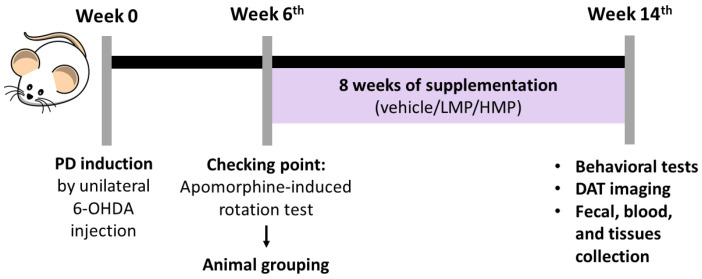
The timeline of the animal experiment.

Sprague Dawley rats (male, aged 8 weeks, weighing 280~300 g) were obtained from Bio-LASCO Taiwan (Taipei, Taiwan) and kept in individual cages at the Laboratory Animal Center of Taipei Medical University (Taipei, Taiwan). Rats were given free access to food and water and were housed under a 12:12 h light–dark cycle, with the temperature controlled to 22~24 °C and 40~60% humidity. After one week of acclimatization, the rats were randomly assigned to the normal healthy control (NC; *n* = 5 rats) group or PD groups (*n* = 15 rats). PD-like motor deficits were induced by an established surgical procedure of unilateral 6-OHDA injection (see Section 2.3). Six weeks after creation of the lesion, the apomorphine-induced rotation test was carried out to confirm the PD model (see Section 2.5.2). Based on the number of contralateral rotations, the rats were allocated into three groups: PD (untreated PD as the negative control; *n* = 5 rats), LMP (low-dose MP; received 30 mg/kg body weight (BW)/day of MP; *n* = 5 rats), and HMP (high-dose MP; received 60 mg/kg BW/day of MP; *n* = 5 rats). Supplementation was administered daily by oral gavage for 8 weeks, beginning the same day as animal grouping. BW and consumption of food and water were monitored weekly. After 8 weeks of supplementation, the apomorphine-induced rotation test, rotarod test, and positron emission tomography/computed tomography (PET/CT) were conducted. Then, rats were euthanized by high-dose anesthesia and cardiac exsanguination. Serum, brain, soleus muscle, and feces were collected for analysis. The timeline of the animal experiment is shown in Figure 1. The procedures of the animal experiments were approved by Taipei Medical University Animal Care and Use Committee under approval no. LAC-2020-0145. All experiments involving animals were conducted in compliance with the code of practice for the care and use of animals for scientific purposes in Taiwan.

### 2.3. Unilateral 6-OHDA Injection

Rats were anesthetized with an intraperitoneal injection of tiletamine-zolazepam (20~40 mg/kg BW) and xylazine (5~10 mg/kg BW). 6-OHDA was unilaterally injected as previously described [40]. Briefly, each anesthetized rat was fixed in the flat skull position using stereotaxic surgery apparatus (David Kopf Instruments, Tujunga, CA, USA) and an electric razor was used to remove the fur. A 0.5~1 cm incision was made to expose the skull. A small burr hole (1 mm) was made in the coordinates of the right medial forebrain bundle [40] to allow the injection of 6-OHDA solution (consisting of 9 μg of 6-OHDA per rat dissolved in 3 μL of a mixture of 0.9% NaCl and 0.02% (*w*/*v*) ascorbic acid) at a constant flow rate via a 10 μL Hamilton syringe attached to an infusion pump. The syringe was left in place for 5 min after the injection and then carefully retracted. The same procedure was performed for the normal control rats (NC group) using the vehicle (3 μL of 0.9% NaCl and 0.02% (*w*/*v*) ascorbic acid mixture). A rotation test with apomorphine induction was performed to confirm the PD rat model at week 6 after 6-OHDA injection. Rats that performed more than seven contralateral rotations/min were considered to be PD rats.

### 2.4. BW Gain, Intake of Food and Water, and Food Conversion Efficiency (FCE)

BW and consumption of food and water were measured every week. BW gain was calculated starting from the day of animal grouping until the end of the supplementation period. FCE was calculated by dividing BW gain by the recorded food intake.

### 2.5. Motor Deficits Assessment

#### 2.5.1. Behavioral Tests

The behavioral tests consisted of the dopamine agonist-induced rotation test and rotarod test. The dopamine agonist-induced rotation test was performed to evaluate dopamine deprivation in the 6-OHDA-injected side by monitoring contralateral rotation. The dopamine agonist apomorphine (Sigma-Aldrich, Darmstadt, Germany) was dissolved in 1% ascorbic acid and 0.9% NaCl and subcutaneously injected into the head of each rat at 0.5 mg/kg BW. The apomorphine injection causes the rats to rotate in the contralateral direction. The contralateral rotation in the apomorphine test was recorded at week 6 after 6-OHDA injection to confirm PD-like motor deficits (more than seven contralateral rotations/min) and after 8 weeks of supplementation to evaluate the effects of treatment. The rotarod test was performed after 8 weeks of supplementation to evaluate locomotor movements and motor coordination. The rotarod test was performed using a rotating rod (Orchid Scientific and Innovative India, Maharashtra, India) at a speed of 10 rpm for a maximum duration of 5 min. The time spent on the rod was recorded.

#### 2.5.2. [^18^F]FE-PE2I PET for Dopamine Transporter (DAT) Imaging

The imaging of DAT was used as a potential surrogate marker for dopaminergic neurons in nigrostriatal pathway. DAT mediates the transport of dopamine and DAT availability is restricted to dopaminergic neurons [41,42]. The analysis of DAT was conducted to further explain the observed motor function. [^18^F]FE-PE2I was prepared as described in a previous study [43]. Rats were anesthetized with tiletamine-zolazepam (20~40 mg/kg BW) and xylazine (5~10 mg/kg BW), then intravenously injected with 200 µL of [^18^F]FE-PE2I via a tail vein and placed in a NanoPET/CT machine (Mediso, Budapest, Hungary). Dynamic [^18^F]FE-PE2I uptake was observed for 60 min. The 3D adjoining Monte Carlo method was used to reconstruct PET images. The counts of an individual image were adjusted for the decay of radioactivity at the injection time and captured images were analyzed. The standard uptake ratio (SUR) of [^18^F]FE-PE21 was determined between the ipsilateral side (injected side) and the contralateral side (non-injected side). Results are presented as ipsilateral/contralateral × 100%.

### 2.6. Assessment of Antioxidant Defense Mechanisms in the Brain and Muscles

#### 2.6.1. Quantification of Reactive Oxygen Species (ROS) Levels

The endogenous antioxidant defense mechanisms in the brain and muscles were assessed by quantifying the production of ROS in the brain and expression of antioxidant genes in the right striatum and left soleus muscle. ROS levels were measured immediately after the samples collection using an ROS fluorometric assay kit for cells and tissues following the manufacturer’s procedures (cat. no. E-BC-K138-F, Elabscience, Houston, TX, USA).

#### 2.6.2. Analysis of Antioxidant Gene Expression by Reverse-Transcription Quantitative Polymerase Chain Reaction (RT-qPCR)

Expressions of antioxidant-related messenger (m)RNAs (*Sod1*, *Sod2*, *Cat*, *Gpx*, and *Nrf2*) were determined in the brain and muscles by RT-qPCR. RNA was extracted from the right striatum and left soleus muscle using Invitrogen™ TRIzol™ reagent (cat. no. 15596018, ThermoFisher Scientific, Waltham, MA, USA) according to the manufacturer’s protocol and complementary (c)DNA was synthesized from the extracted RNA (1 µg) by the RT principle using the iScript™ cDNA synthesis kit (Bio-Rad, Hercules, CA, USA). RT-qPCR was performed using the primers shown in Table S1 on a LightCycler^®®^ 96 System (Roche, Mannheim, Germany). Gene expression levels were quantified using the 2^−∆∆CT^ method, normalized to β-actin, and expressed relative to the NC group.

### 2.7. Evaluation of Systemic Antioxidant Activity and Inflammatory Markers Using an Enzyme-Linked Immunosorbent Assay (ELISA)

Systemic antioxidant activity was evaluated by measuring total superoxide dismutase (SOD) activity in serum using an ELISA kit (cat. no. E-BC-K020-M, Elabscience, Houston, TX, USA). Inflammatory markers in the circulatory system were evaluated by measuring the inflammatory cytokines interleukin (IL)-1β, IL-6, and tumor necrosis factor (TNF)-α in plasma using ELISA kits (cat. no. E-EL-R0674; cat. no. E-EL-R0012; cat. no. E-EL-R0015; E-EL-R2856, Elabscience). All analytical procedures followed the manufacturer’s protocol.

### 2.8. Analysis of Mitochondrial Function and Mitochondrial Gene Expression in the Brain and Muscles

#### 2.8.1. Seahorse Bioscience XFe24 Extracellular Flux Analysis

Mitochondrial function was assessed in real time using a Seahorse XFe24 extracellular flux analyzer (Seahorse Bioscience, North Billerica, MA, USA). Measurements of the basal oxygen consumption rate (OCR) and basal extracellular acidification rate (ECAR) were quantified in the lesioned side of the brain and left soleus muscle, as described in a previous study [44]. Briefly, the freshly isolated right striatum and left soleus muscle were homogenized and added to an islet capture 24-well microplate provided by Seahorse Bioscience (cat. no. 101122-100). The assay XF calibration solution (cat. no. 100867-000, Seahorse Bioscience) was added to a dedicated cartridge plate from Seahorse Bioscience (cat. no. 100867-100) and incubated in at 37 °C in carbon dioxide-free incubator for 24 h. As in the sample cartridge, isolated tissues were positioned at the base of the microplate and enclosed with a ring screen. Seahorse XF Assay medium (525 µL; cat. no. 102365-100, Seahorse Bioscience) was added to each well and the sample cartridge was incubated at 37 °C in a carbon dioxide-free incubator for 20 min and placed into the analyzer instrument. Measurements of the real-time basal OCR and ECAR were repeated for up to five cycles. After the real-time basal OCR and ECAR measurements, the tissue samples were removed from the microplate analyzed using the BSA-Bradford protein quantification assay (Sigma-Aldrich, Darmstadt, Germany) to determine the protein concentration.

#### 2.8.2. RT-qPCR Analysis of Mitochondrial Gene Expression

The mitochondrial (mt)DNA copy number, including *Nd1* and *Atp6*, and mitochondrial biogenesis-related mRNA expression (*Pgc1a*, *Nrf1*, and *Tfam*) were analyzed in the right striatum and left soleus muscle using an RT-qPCR method using the primers shown in Table S1. RNA extraction, cDNA synthesis, qPCR, and quantification were performed using the protocols described in Section 2.6.2.

### 2.9. Evaluation of Fecal Microbiota Composition

#### 2.9.1. Bacterial DNA Isolation and 16S rRNA Amplicon Sequencing

Fresh fecal samples from individual rats were collected the day after the final supplementation by abdominal massage to accelerate the defecation process. DNA was isolated using a QIAamp Fast DNA Stool Mini Kit (Qiagen, Germantown, MD, USA) following the manufacturer’s protocol. The isolated DNA was assessed with a NanoDrop 2000 spectrophotometer to confirm the target OD 260/280 ratio ranged from 1.8~2.0. Amplification of the standard V3~V4 region of the 16S ribosomal (r)RNA gene was conducted by PCR. AMPure XP magnetic beads (Beckman Coulter, Brea, IN, USA) were used to purify the PCR product, then the Nextera XT Index Kit (Illumina, San Diego, CA, USA) was used to label the amplicons. The product quality of the amplicons was assessed using a Fragment Analyzer (Advanced Analytical, Ankeny, IA, USA), then the amplicons were quantified using the Qubit dsDNA HS assay ki (Life Technologies, Pleasanton, CA, USA), following the manufacturers’ procedures. The libraries were constructed by sequencing using a MiSeq Reagent Kit V3 on a MiSeq (Illumina) to obtain paired-end reads (2 × 300 nt) over 600 cycles.

#### 2.9.2. Bioinformatic Analysis

The bioinformatics analysis consisted of raw reads of quality filters, classification of operational taxonomic units (OTUs), diversity profile analysis, and linear discriminant analysis (LDA) of effect size (LEfSe). Raw paired-end reads were trimmed, filtered by quality trimming, short read lengths were discarded and chimeras were removed, and the filtered reads were compared to the Greengene database. Reads that showed ≥97% similarity were classified into the same OTU. The BaseSpace Ribosomal Database Project (RDP) classifier was also used to analyze raw paired-end reads. OTUs were analyzed by relative abundances, α-diversities were analyzed using the Shannon index, and β-diversities were analyzed by principal component analysis (PCoA) based on the Bray–Curtis index using BaseSpace (Illumina), CLC Genomics Workbench 21 with the Microbial Genomics Module (Qiagen) and GraphPad Prism 8. Specific microbial enrichments between groups were identified by further analysis of OTU table via LEfSe (α = 0.05, Kruskal–Wallis and Wilcoxon tests), with 2 set as the effect size threshold and LDA scores were analyzed to identify significant taxa. Relative abundances of specific genera and species were further analyzed by parametric analysis using unpaired *t*-tests or non-parametric analysis with the Mann–Whitney test to investigate the differences between the untreated PD group and supplementation group.

### 2.10. Statistical Analysis

Results are presented as the average ± standard error of mean (SEM). GraphPad Prism 8 statistical software was used to analyze the differences between groups. Significance was defined as a *p*-value of <0.05. Differences were evaluated using a one-way analysis of variance (ANOVA) followed by Tukey’s honest significant difference (HSD) or Dunnett’s post hoc test. Spearman’s correlation analysis was conducted to analyze the correlations between motor function and antioxidant-related parameters, between bacteria and motor functions, and between bacteria and antioxidants.

## 3. Results

### 3.1. LMP and HMP Supplementations Do Not Affect BW Gain, Food Intake, FCE, or Water Intake

The BW gain and consumption of food and water by the rats were monitored from creation of the 6-OHDA lesion until the end of the 8-week supplementation period (Table 1). BW increased in all groups during the experimental period. There were no significant differences in BW gain or the consumption of food and water among groups. Additionally, all groups exhibited similar FCEs. These data suggest that the supplementation did not affect BW gain, intake of food or water, or the FCE.

### 3.2. Supplementation with LMP and HMP Improves Motor Function in a PD-like Condition

**Figure 2:**
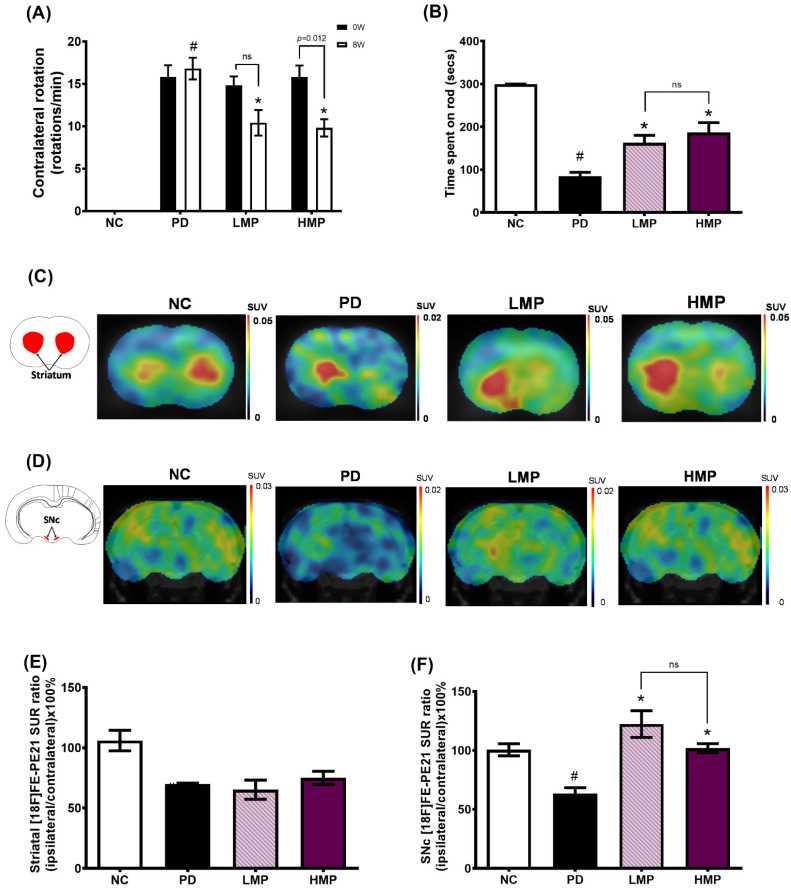
Effects of mangosteen pericarp (MP) extract supplementation on motor function. (**A**) Number of contralateral rotations recorded in the apomorphine-induced rotation test at week 0 and week 8 of supplementation. (**B**) Evaluation of motor coordination and balance skills in the rotarod test after 8 weeks of supplementation. (**C**) Representative images of the standard uptake ratio (SUR) of [^18^F]FE-PE21 in the striatum, illustrating the dopamine transporter (DAT) binding activity. (**D**) Representative images of the SUR of [^18^F]FE-PE21 in the substantia nigra pars compacta (SNc). (**E**) Quantitation of the SUR ratio in the striatum. (**F**) Quantitation of the SUR ratio in the SNc. Data are average ± SEM for *n* = 5 rats/group and were compared using one-way ANOVA with Tukey’s post hoc test; # *p* < 0.05 for Parkinson’s disease (PD) group vs. normal control (NC) group. * *p* < 0.05 for low-dose mangosteen pericarp (LMP) and high-dose mangosteen pericarp (HMP) groups vs. PD group. ns, not significant.

The apomorphine-induced rotation test and rotarod test were performed to evaluate the effects of MP extract on behavior and motor function in the rat model of PD. The apomorphine-induced rotation test was conducted before and after MP extract supplementation to assess the severity of denervation in the motor control basal ganglia circuit. Apomorphine, which is a dopamine receptor agonist, stimulates both classes of dopamine receptors (D1, D2), thus induces contralateral response of 6-OHDA-lesioned animals [45]. In this study, we first conducted the apomorphine-induced rotation test at 3 weeks after 6-OHDA lesion, however, the contralateral rotations were too few to be included in our study. Therefore, we conduct the second apomorphine-induced rotation test at 6 weeks after 6-OHDA lesion. At week 6 after creation of the 6-OHDA lesions (week 0), all PD groups exhibited an average of 15 contralateral rotations/min, whereas contralateral rotation was not observed in the sham NC group; these findings confirmed establishment of the animal model of PD (Figure 2A). After 8 weeks of supplementation (week 8), the untreated PD group still exhibited a significantly higher number of contralateral rotations than the healthy NC group. In contrast, the groups treated with LMP and HMP for 8 weeks exhibited a significantly lower number of contralateral rotations compared to the untreated PD group. This indicates that both the low and high doses of MP extract potentially protected dopaminergic neurons in the motor control basal ganglia circuit.

We also evaluated motor coordination and balance skills after 8 weeks of supplementation by performing the rotarod test (Figure 2B). Compared to the healthy group, the untreated PD group spent a significantly shorter time on the rod, indicating motor dysfunction. Supplementation with both the low and high doses of MP for 8 weeks attenuated the motor dysfunction in PD rats, as indicated by a longer time spent on the rod compared to the untreated PD group. There was no significant difference between the LMP and HMP groups in the rotarod test, which suggests that both doses of MP improved motor coordination and balance skills in a PD-like condition.

To further evaluate the motor function, we investigated DAT binding activity in the striatum and SNc using [^18^F] FE-PE21 PET (Figure 2C–F); the radioligand [^18^F]FE-PE21 specifically binds to DAT, which is only expressed by live dopaminergic neurons [46]. The binding process produces light signal that is converted into an electrical signal, which is used to determine the radioligand uptake value that is used to quantify the numbers of active dopaminergic neurons in the striatum and SNc [47]. A lower membrane DAT expression on presynaptic terminals may possibly reflect striatal dopamine terminal loss and it shows indirect proportion to the magnitude of the depletion of nigral cells [48].

In the striatum, the groups lesioned with 6-OHDA showed lower [^18^F] FE-PE21 SUR ratios compared to the healthy NC group (Figure 2E). Although not statistically significant, the striatal uptake ratio of the HMP group was slightly higher than that of the untreated PD group. However, the untreated PD group exhibited a significantly lower [^18^F] FE-PE21 SUR ratio in the SNc than the healthy NC group (Figure 2F). In contrast to the untreated PD group, the groups treated with LMP and HMP exhibited significantly higher SURs in the SNc, with the LMP group showing a slightly higher SUR than the HMP group. A higher uptake ratio indicates higher DAT binding activity, suggesting a possible increase in the number of active dopaminergic neurons. Taken together with the motor function results, the PET results indicate that supplementation with the MP extract, at either a low or high dose, preserved active dopaminergic neurons in the motor control basal ganglia circuit and may have improved motor function in this PD-like rat model.

### 3.3. Supplementation with LMP and HMP Decreases Brain ROS Levels, Increase Expression of Antioxidant Genes, and Reduce Plasma Inflammatory Cytokines

To explore the possible mechanisms related to the neuroprotective effects and improved motor function observed in the LMP and HMP groups, we quantified the levels of ROS levels in the brain, antioxidant-related mRNA expression in the brain and muscle, total SOD levels in serum, and the levels of inflammatory cytokines in plasma. 

**Figure 3:**
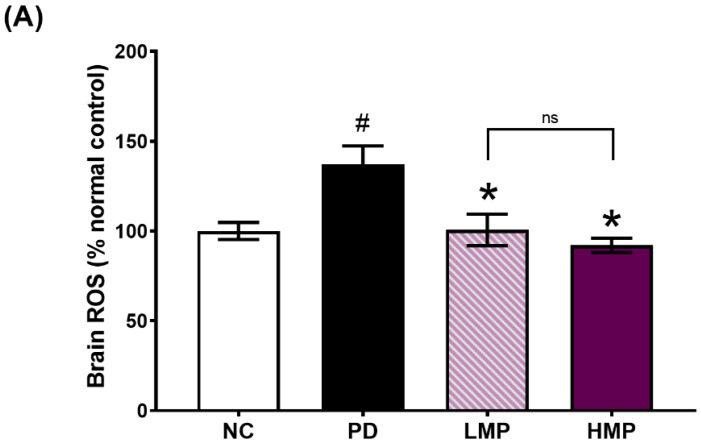

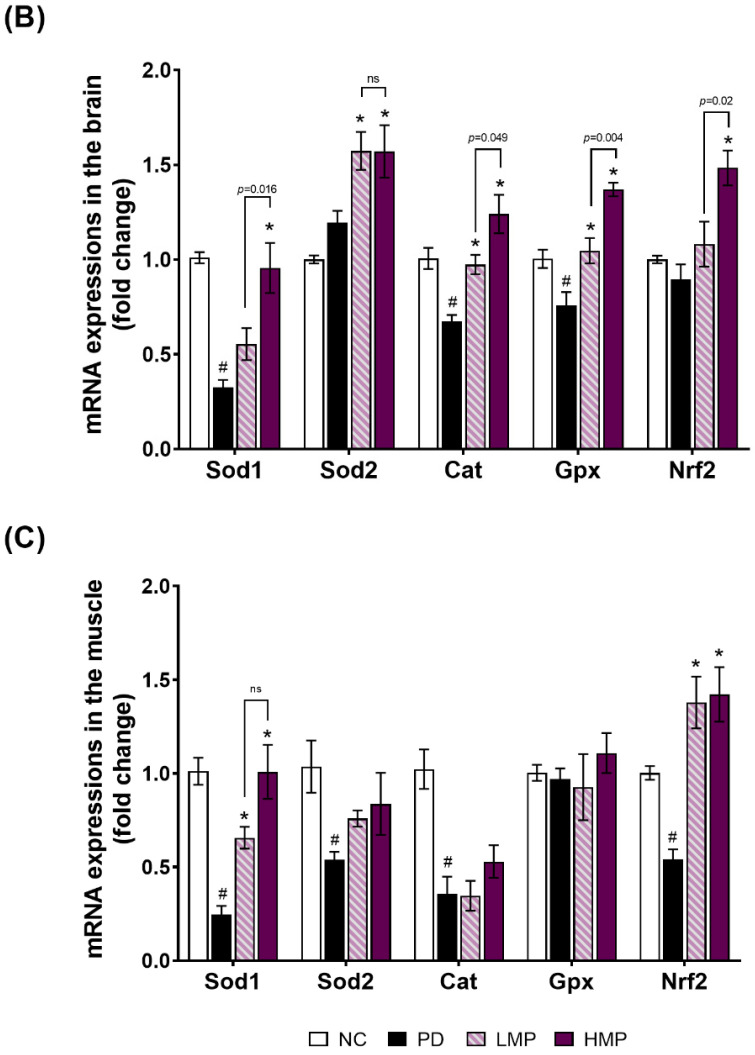
Levels of ROS in the brain (**A**) and antioxidant-related gene expression in (**B**) the brain and (**C**) muscle after 8 weeks of supplementation in the rat model of PD. All data are average ± SEM for *n* = 5 rats/group and were analyzed using one-way ANOVA with Tukey’s post hoc test. # *p* < 0.05 for the Parkinson’s disease (PD) group vs. normal control (NC) group, * *p* < 0.05 for the low-dose mangosteen pericarp (LMP) and high-dose mangosteen pericarp (HMP) groups vs. the PD group. *Sod*, superoxide dismutase; *Cat*, catalase; *Gpx*, glutathione peroxidase; *Nrf2*, nuclear factor-erythroid factor 2-related factor 2. ns, not significant.

We analyzed the levels of ROS and the expression of *Sod1*, *Sod2*, *Cat*, *Gpx*, and *Nrf2* in the brain (Figure 3A,B). The levels of ROS in the brain were significantly higher in the untreated PD group than the healthy NC group. In contrast, the groups supplemented with LMP and HMP had significantly lower levels of ROS level in the brain. Moreover, the reductions in ROS in the supplemented groups were accompanied by increased expression of *Sod1*, *Sod2*, *Cat*, and *Gpx* in the brain.

**Figure 4:**
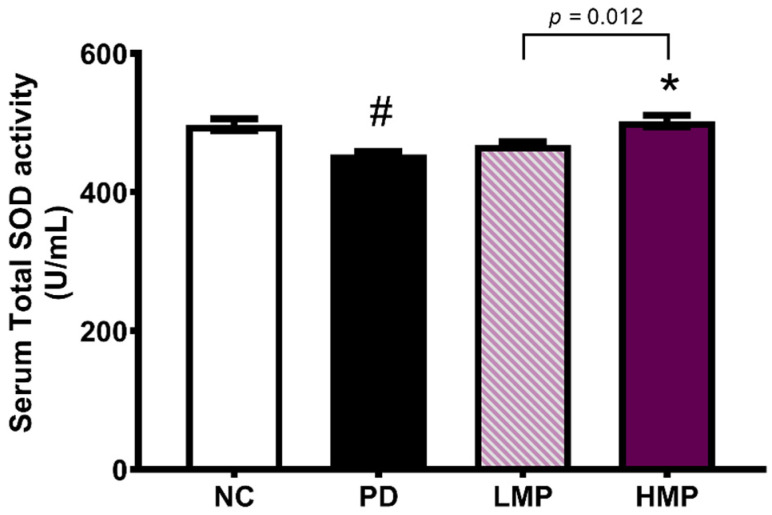
Serum levels of total superoxide dismutase (SOD) after 8 weeks of supplementation in the rat model of PD. All data are average ± SEM for *n* = 5 rats/group and were analyzed using one-way ANOVA with Tukey’s post hoc test. # *p* < 0.05 for the Parkinson’s disease (PD) group vs. normal control (NC) group; * *p* < 0.05 for low-dose mangosteen pericarp (LMP) and high-dose mangosteen pericarp (HMP) groups vs. the PD group.

To investigate whether a link exists between the observed improvement in motor function and the antioxidant capacity of skeletal muscle, we next analyzed the mRNA expression of endogenous antioxidants (*Sod1*, *Sod2*, *Cat*, and *Gpx*) and an antioxidant-related transcription factor (*Nrf2*) in the soleus muscle (Figure 3C). After the 6-OHDA injection, the untreated PD group exhibited significantly lower expression of *Sod1*, *Sod2*, *Cat*, and *Nrf2* in the muscle compared to the healthy NC group. However, supplementation of PD rats with MP led to higher expression of these antioxidant-related genes in the muscle, with statistically significant increases in *Sod1* and *Nrf2*. Moreover, the HMP group exhibited a significantly higher serum level of total SOD compared to the PD group (Figure 4), suggesting that high-dose MP might offer more-potent antioxidant activity than low-dose MP.

Next, we analyzed the correlations between the motor function and antioxidant parameters (Table 2). The expression levels of *Sod* and *Cat* in the brain and muscle correlated positively with locomotor function. In contrast, the levels of ROS in the brain correlated negatively with locomotor function. This further indicates the potential therapeutic ability of MP to delay motor deficits by improving the endogenous antioxidative defense mechanisms. In addition, the HMP group exhibited a significantly higher level of *Nrf2* expression compared to the PD group, which suggests that the high-dose MP exerted more potent antioxidant capacity than the low-dose MP.

Additionally, at the systemic level, the induction of PD via 6-OHDA injection appeared to trigger an inflammatory response in the blood circulatory system, as observed by elevated plasma levels of IL-1β, IL-6, and TNF-α in the untreated PD group (Table 3). In contrast, both groups supplemented with MP exhibited significantly lower levels of IL-1β, IL-6, and TNF-α in plasma than the PD group.

### 3.4. Effects of LMP and HMP on Mitochondrial Function, mtDNA Copy Number and Mitochondrial Biogenesis in Muscle

**Figure 5:**
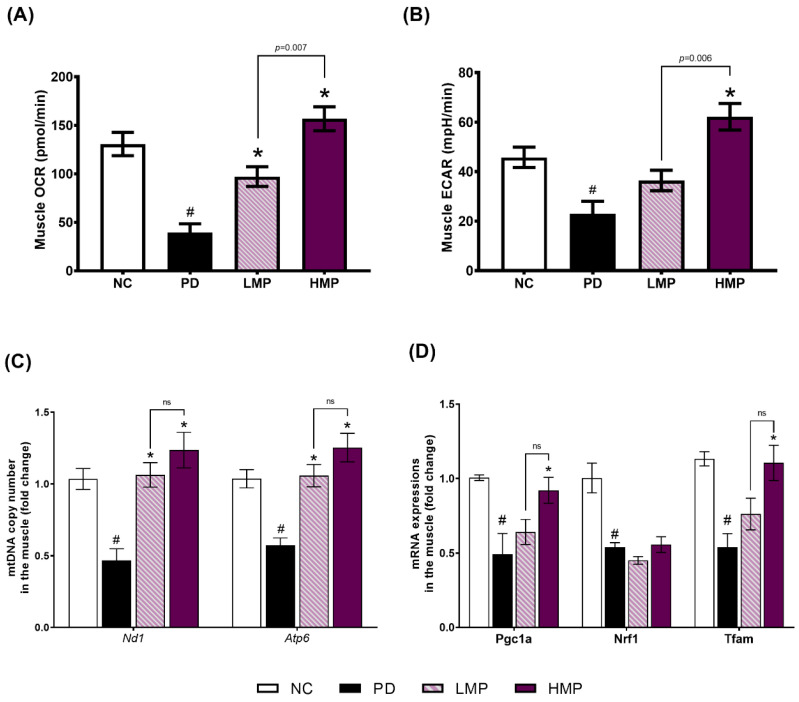
Comparison of mitochondrial function and mitochondria-related mRNA expression in muscles after 8 weeks of supplementation in the rat model of PD. (**A**) The basal oxygen consumption rate (OCR) was quantified to evaluate mitochondrial respiration. (**B**) Basal extracellular acidification flux (ECAR) was used to evaluate the rate of glycolysis. (**C**) mtDNA copy numbers (*Nd1* and *Atp6*). (**D**) Expression of mitochondrial biogenesis-related mRNAs: PPARG coactivator 1 alpha (*Pgc1a*), nuclear respiratory factor 1 (*Nrf1*), mitochondrial transcription factor A (*Tfam*). Results are average ± SEM for *n* = 5 rats/group and were analyzed using one-way ANOVA with Tukey’s post hoc test. # *p* < 0.05 for the Parkinson’s disease (PD) group vs. the normal control (NC) group. * *p* < 0.05 for the low-dose mangosteen pericarp (LMP) and high-dose mangosteen pericarp (HMP) groups vs. the PD group. ns, not significant.

To investigate the possible linkage between mitochondrial function and the observed improvement in motor function due to the MP extract, we analyzed the OCR and ECAR levels in skeletal muscle. Compared to the healthy NC group, the untreated PD group exhibited significantly lower OCR and ECAR levels in the soleus muscle (Figure 5A,B). However, LMP and HMP supplementation attenuated these decreases in PD rats. The group treated with HMP showed the highest muscle OCR and ECAR levels. This data indicates that both supplementation doses had beneficial impacts on muscle mitochondrial function, but that the higher dose of MP more potently improved the mitochondrial function and rate of glycolysis in the skeletal muscle of PD rats.

Following the assessment of OCR and ECAR levels in skeletal muscle, we next assessed the mtDNA copy number and expression of genes responsible for mitochondrial biogenesis. Consistent with the results observed in the brain (Figure S2), injection of 6-OHDA reduced the mtDNA copy number and impaired mitochondrial biogenesis in skeletal muscle: the untreated PD group exhibited significantly lower mtDNA copy numbers of *Nd1* and *Atp6* (Figure 5C), accompanied by lower expression of genes involved in the biogenesis of mitochondria (*Pgc1a*, *Nrf1*, and *Tfam*) (Figure 5D) compared to the NC group. In contrast, both groups supplemented with MP extract for 8 weeks had significantly higher mtDNA copy numbers than the untreated PD group. Furthermore, the HMP group showed significantly higher *Pgc1a* and *Tfam* expression than the PD group, indicating restoration of mtDNA copy number. These results collectively indicate that the improvement in motor function observed in the supplemented groups might not solely be due to preservation of SNc dopaminergic neurons, but also to maintenance of mitochondrial function, the mtDNA copy number, and mitochondrial biogenesis in skeletal muscles.

### 3.5. Long-Term Supplementation with LMP and HMP Alters the Gut Microbiota Profile in the PD-like Rat Model

**Figure 6:**
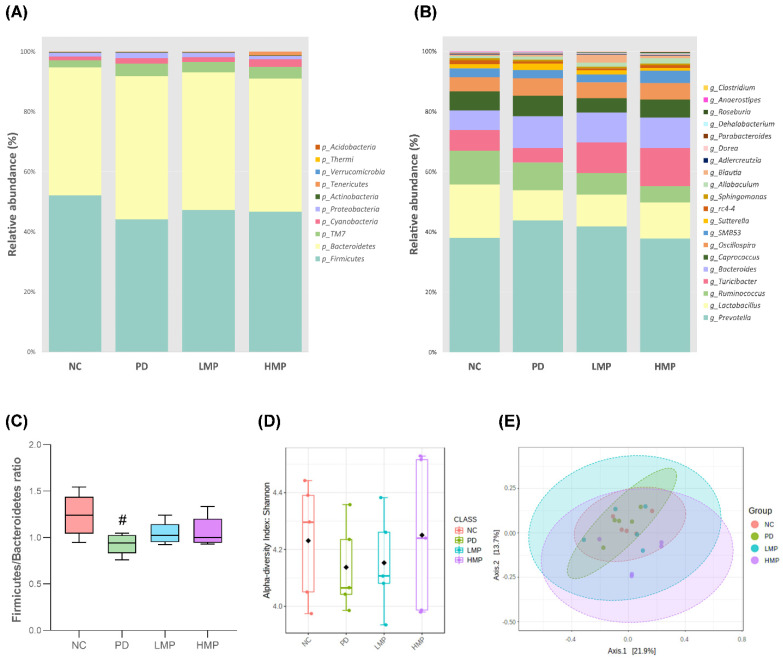
Comparison of the relative bacterial abundances and analysis of bacterial diversity based on 16S sequencing analysis of fecal samples after 8 weeks of supplementation in the rat model of PD. (**A**) Distribution of the top ten most abundant bacteria at the phylum level across all groups. (**B**) Distribution of the top twenty most abundant bacteria at the genus level across all groups. (**C**) Firmicutes to Bacteroidetes ratios of each group. # *p* < 0.05 for the Parkinson’s disease (PD) group vs. the normal control (NC) group. (**D**) Analysis of alpha diversity based on the Shannon index. (**E**) Analysis of beta-diversity based on principal coordinates analysis (PCoA) of the Bray–Curtis index. The ellipses illustrate 95% confidence intervals for the normal control (NC) versus Parkinson’s disease (PD) versus low-dose mangosteen pericarp (LMP) versus high-dose mangosteen pericarp (HMP).

The gut microbiota composition has been reported to be altered under PD conditions. In our previous study, we found that induction of a rat model of PD by unilateral 6-OHDA injection replicated the gut dysbiosis that occurs under PD conditions. In this study, we assessed the fecal microbiota profiles to investigate whether LMP or HMP mediate the observed changes in PD parameters by altering the microbiota composition in the gastrointestinal tract. After 8 weeks of supplementation, the relative abundances differed between groups. At the phylum level, the top ten phyla across all groups were Firmicutes (40~48%), Bacteroidetes (39~44%), TM7 (2~4%), Cyanobacteria (1~3%), Proteobacteria (1~2%), Actinobacteria (~0.2%), Tenericutes (0.1~1%), Verrucomicrobia (0~0.02%), Thermi (<0.01%), and Acidobacteria (<0.01%; Figure 6A). Compared to the healthy NC group, the untreated PD group showed a significant decrease in Firmicutes (*p* = 0.004) and trends towards increases in Bacteroidetes and Proteobacteria. Compared to the untreated PD group, the LMP or HMP groups exhibited trends towards an increase in Firmicutes (PD vs. LMP; *p* = 0.038 and PD vs. HMP; *p* = 0.337). Supplementation with HMP significantly suppressed Proteobacteria colonization under the PD-like condition (*p* = 0.022). Additionally, the HMP group showed notable increases in Cyanobacteria and Tenericutes. Furthermore, the Firmicutes to Bacteroidetes ratio was significantly lower in the untreated PD group than the NC group, whereas the supplementation groups tended to have higher Firmicutes to Bacteroidetes ratios than the PD group (Figure 6C).

The 20 genera with the highest relative abundances across the groups are illustrated in Figure 6B. At the genus level, *Prevotella* (15~19%), *Lactobacillus* (4~9%), *Ruminococcus* (2~5%), *Turicibacter* (2~5%), *Bacteroides* (3~5%), *Caprococcus* (2~3%), *Oscillospira* (~2%), *SMB53* (1~2%), *Suterella* (0~1%), and *rc_4* (0.3~0.6%) were the ten most abundant genera in all groups. Injection of 6-OHDA in the PD group was associated with alterations in the gut microbiota composition. Specifically, trends towards increases in *Prevotella*, *Bacteroides*, and *Sutterella* were observed in the untreated PD group compared to the NC group. The PD group also exhibited declines in the relative abundance of *Lactobacillus* and *Turicibacter*. The ability of the LMP and HMP to reshape the microbiota profile was also observed at the genus level. In particular, HMP supplementation tended to decrease *Prevotella* and *Sutterella* and increase *Lactobacillus* and *Turicibacter*.

Moreover, we compared the alpha and beta diversities of the groups. According to the Shannon index, the PD group exhibited lower alpha diversity than the NC group, but this difference was not statistically significant. Alpha-diversity was not altered in either supplementation group as compared to the PD group (Figure 6D). The PD and NC groups showed no significant differences in beta diversity based on PCoA of the Bray–Curtis index. However, the HMP group exhibited distinct microbiota communities compared to the untreated PD group (Figure 6E).

**Figure 7:**
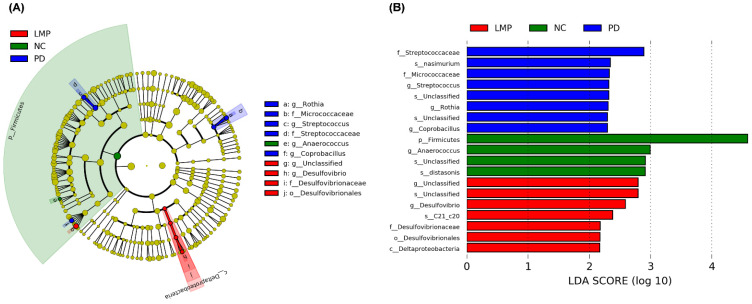
Linear discriminant analysis (LDA) effect size (LEfSe) of fecal microbiota enrichment in the normal control (NC), Parkinson’s disease (PD), low-dose mangosteen pericarp (LMP), and high-dose mangosteen pericarp (HMP) groups after 8 weeks of supplementation. (**A**) The circles of the phylogenetic tree cladogram demonstrate the classification of the fecal microbiota from the phylum to genus level. The diameter of the circles illustrates relative abundance. The typescripts A to J in A represent 10 different taxa. (**B**) LDA scores for differentially abundant taxa between the NC, PD, LMP, and HMP groups. Taxa with an LDA score >2 (log 10) were included in the LEfSe analysis. There was no distinct taxonomic enrichment between the HMP group and other groups; thus, the HMP group is not shown in the phylogenetic tree cladogram. C, class; F, family; G, genus; O, order; P, phylum.

Next, we analyzed the richness and patterns of abundance of specific taxa using LEfSe to investigate the effects of supplementations on the fecal microbiota composition in more detail (Figure 7). The NC group showed the highest abundance of the phylum Firmicutes. In contrast, the PD group showed enrichment of the genera *Streptococcus*, *Rothia* and *Coprobacillus.* Interestingly, the LMP group showed enrichment of the family Desulfovibrionaceae and genus *Desulfovibrio.* The HMP group exhibited no distinct taxonomic enrichment compared to the other groups.

**Figure 8:**
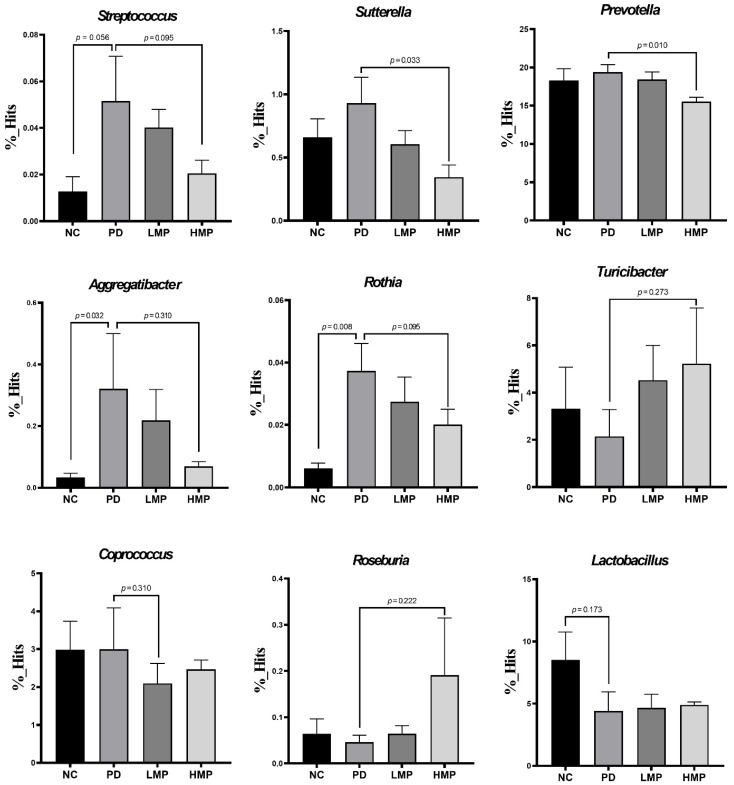
Analysis of the abundance of specific bacteria at the genus level after 8 weeks of supplementation in the low-dose mangosteen pericarp (LMP) and high-dose mangosteen pericarp (HMP). Results are average ± SEM for *n* = 5 rats/group and were analyzed using the unpaired *t*-test or Mann–Whitney test.

**Figure 9:**
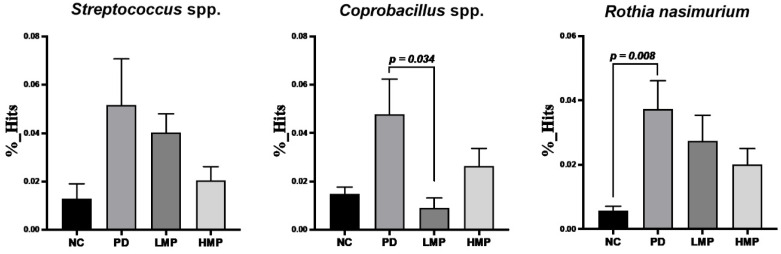
Comparison of the abundance of specific bacterial species identified in rats with 6-OHDA-induced Parkinson’s disease after 8 weeks of supplementation with the low-dose mangosteen pericarp (LMP) and high-dose mangosteen pericarp (HMP). Results are average ± SEM for *n* = 5 rats/group and were analyzed using unpaired *t*-tests.

Following the LEfSe analysis, we further analyzed changes in specific genera and species, as shown in Figure 8 and Figure 9, respectively. Specifically, trends towards increases in *Streptococcus, Sutterella*, *Prevotella*, *Aggregatibacter*, and *Rothia* were observed in the untreated PD group compared to the NC group. Concomitantly, slight reductions in *Turicibacter*, *Roseburia*, and *Lactobacillus* were also found in the untreated PD group. In contrast, the HMP group exhibited significant decreases in *Prevotella* and *Sutterella*. Although not significant, the relative abundances of *Streptococcus, Sutterella*, *Aggregatibacter*, and *Rothia* decreased while the relative abundances of *Turicibacter*, *Roseburia*, and *Lactobacillus* were relatively increased in the HMP group compared the untreated PD group. The microbiota-reshaping effects of HMP were also noted at the species level, with relatively lower abundance of *Streptococcus* spp., *Coprobacillus* spp., and *Rothia nasimurium* observed in the HMP group.

Last, we investigated the associations between specific bacterial taxa (at the genus and species level) and parameters related to the progression of PD, including motor function, antioxidant capacity, muscle mitochondria-related gene expression, and plasma inflammatory cytokines (Table 4). Bacterial genera that were abundant in the untreated PD group, namely *Sutterella*, *Rothia*, *Streptococcus*, and *Aggregatibacter*, were negatively correlated with antioxidant-related gene expression and positively correlated with the levels of plasma pro-inflammatory cytokines. *Turicibacter*, which was abundant in the HMP group, was positively correlated with *Gpx* expression in the brain. Importantly, *Streptococcus* and *Rothia* were negatively correlated with locomotor function. Taken together, the fecal microbiota analyses suggest that supplementation with MP, in particular HMP, rebalanced the fecal microbiota profile of gut dysbiosis in this 6-OHDA-induced PD-like rat model. Specifically, HMP supplementation prevented increases in Proteobacteria at the phylum level and *Rothia*, *Suterella*, *Prevotella*, *Streptococcus*, and *Aggregatibacter* at the genus level and prevented decreases in Firmicutes at the phylum level and *Roseburia*, *Lactobacillus*, *Turicibacter* at the genus level. Moreover, these changes in the fecal microbiota profile may be related to the antioxidant capacity exerted by MP.

## 4. Discussion

Oxidative stress and gut dysbiosis have been suggested to play critical roles in the deterioration of dopaminergic neurons and motor function in PD. Most of the available therapies, such as L-DOPA drugs, only address the motor symptoms caused by the lack of dopamine. Therefore, new therapies that not only alleviate the motor symptoms, but also attenuate the pathogenic mechanisms, such as oxidative stress and gut dysbiosis, are urgently needed. This study shows that MP extract directly acts as an antioxidant to counterbalance excessive production of free radicals and may also indirectly increase antioxidant capacity by rebalancing the gut microbiota, and the resulting enhanced anti-inflammatory capacity and restoration of mitochondrial function attenuate the motor deficits and slow down the progression of PD.

Supplementation with polyphenol-rich MP extract for 8 weeks at both a low dose (LMP) or a high dose (HMP) attenuated the progression of PD in our 6-OHDA-induced PD-like rat model, as indicated by significant improvements in motor function (Figure 2A,B) and DAT binding activity in the SNc (Figure 2F). These effects may be mainly derived from a cascade of cellular mechanisms involving the antioxidant defense system and gut microbiota. The antioxidant compounds in MP might act directly on the cellular antioxidant defense system to reduce oxidative stress in the brain and increase the endogenous expression of antioxidant genes in the brain, including *Sod1*, *Sod2*, *Cat*, *Gpx*, and *Nrf2*. As a result, MP reduced the levels of ROS in the brain (Figure 3A,B). Moreover, MP supplementation also attenuated 6-OHDA-induced decrease in total SOD in the serum (Figure 4) and suppressed production of inflammatory cytokines (i.e., IL-1β, IL-6, TNF-α) in plasma (Table 3), which implies MP may have similar effects in the brain. Furthermore, the MP extract elevated the antioxidant capacity (Figure 3B) and prevented the 6-OHDA-induced impairments to mitochondrial biogenesis in muscle, which consequently improved muscle mitochondrial function and energy metabolism (Figure 5) and may further prevent motor dysfunction.

**Figure 10:**
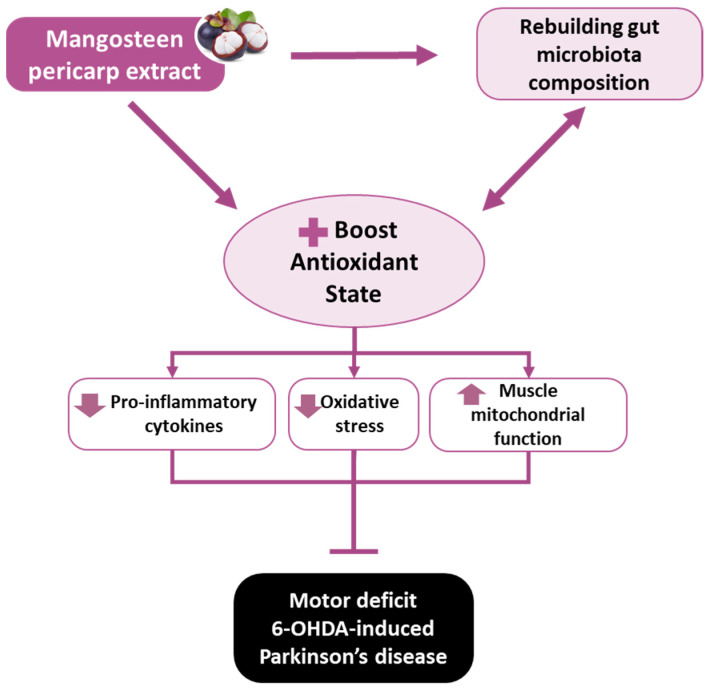
The proposed mechanism of action by which mangosteen pericarp extract delays motor deficit in 6-OHDA-induced PD.

Moreover, MP extract may also indirectly improve the cellular antioxidant defense system through fecal microbiota alterations (Figure 6, Figure 7, Figure 8 and Figure 9). Some of the alterations observed in the abundance of fecal bacteria positively correlated with enhanced antioxidant capacity in the brain and muscles. The correlation between the fecal microbiota and antioxidant capacities in the brain and muscles indicate a potentially important role for the gut microbiota profile in the progression of PD. Additionally, the abundance of several bacterial genera that positively correlated with the levels of pro-inflammatory cytokines were suppressed by MP. This strongly suggests that the mechanisms of action MP extract might be initiated by reshaping the gut microbiota profile, which in turn regulates antioxidative and inflammatory responses at the systemic level and, as a consequence, MP affected the antioxidant capacity and energy metabolism in the brain and muscles. Ultimately, the MP extract protected active dopaminergic neurons and attenuated motor deficits. The proposed mechanisms of action by which MP extract delayed the motor deficits in 6-OHDA-induced PD are presented in Figure 10.

Oxidative stress has long been associated with the advancement of neurodegeneration in PD. Aging, neurotoxins, and neuroinflammation are known to induce oxidative stress during the progression of PD [49,50]. Elevated oxidative stress has been reported to result in mitochondrial damage in patients with PD [8,10,51] and the subsequent impairments to mitochondrial function lead to excessive ROS production [12]. Under normal conditions, ROS can be neutralized by endogenous antioxidants (e.g., SOD, catalase, and GPx). However, these endogenous antioxidants are depleted in PD [52,53]. Specifically, several lines of evidence indicate SOD1 expression and activity is reduced in both in vitro and clinical studies of PD [54,55,56,57], and copper deficiency has been suggested as one possible explanations for these changes [58]. Herein, we revealed that supplementation of MP extract elevated the endogenous expression levels of antioxidant-related genes (*Sod1*, *Sod2*, *Gpx*, and *Cat*; Figure 3) and reduced the levels of ROS in the brain (Figure 3A), and also preserved dopaminergic neurons in the SNc (Figure 2F). Importantly, supplementation with the higher dose of MP (HMP) stimulated the expression of *Nrf2*, a key regulator of antioxidant and redox status.

Polyphenol-rich MP extract has been reported to possess antioxidant [34] and anti-inflammatory properties [35] as it contains various bioactive compounds, including xanthones (e.g., α-mangostin, β-mangostin, and γ-mangostin), anthocyanins, and proanthocyanidins [32,33]. The xanthones contained in MP were demonstrated to exert potent antioxidant capacity to counterbalance the excessive ROS production during the PD-like pathogenesis [34,59,60,61]. MP was also shown to enhance protein expression of NRF2 [62], which upregulates secretion of endogenous antioxidant enzymes including SOD, CAT, and GPx [63,64]. Furthermore, the reduction in oxidative stress conferred by the antioxidant activity of MP was shown to inhibit mitochondrial-dependent apoptosis of dopaminergic neurons [59,60]. The antioxidant properties of α-mangostin were found to alleviate neuroinflammation [35,65], which may prevent dopaminergic neuronal loss in PD. In addition, the xanthones contained in MP were reported to suppress monoamine oxidase (MAO) activity [66,67,68], which produces ROS by oxidation of neurotransmitters such as dopamine [69,70].

The oxidative stress observed in PD is commonly followed by inflammation. Studies have reported increased numbers of activated microglia cells and astrocytes, along with higher levels of proinflammatory cytokines, in the brain of patients with PD [71]. Importantly, some studies suggest that the inflammation is not only present in the brain, but also in the peripheral blood [71,72]. In this study, we showed that LMP and HMP reduced the levels of pro-inflammatory cytokines (i.e., IL-1β, IL-6, and TNF-α) in plasma; these reductions were associated with increased total SOD activity in serum. This suggests that antioxidant activity of MP enhanced the endogenous antioxidant activity, which in turn reduced the levels of pro-inflammatory cytokines in the blood. These results also indirectly suggest that the antioxidant activity of MP might potentially reduce neuroinflammation in the brain.

Prolonged exposure to free radicals can also exacerbate the mitochondrial dysfunction in PD. Mitochondrial dysfunction has been documented in the SNc region of the brain in patients with PD [2,15] and is associated with depletion of mitochondrial proteins (i.e., complexes I, II, and IV) [8,9,10]. The depletion of these protein complexes impairs the electron transport reaction during oxidative phosphorylation, which consequently leads to decreased energy production and excessive ROS production [51] and creates a vicious cycle that continually damages mitochondrial function [18]. In this study, we observed that supplementation with HMP for 8 weeks increase the expression of antioxidant-related genes in the brain and slightly increased the oxygen consumption rate (OCR) in the brain, indicating HMP may potentially improve mitochondrial function (Figure S2). Our results are in line with Hao et al. [61], who reported α-mangostin treatment improved mitochondrial function, increased ATP production, and also prevented dopaminergic neuronal death in rotenone-induced neuroblastoma cells.

Previous studies reported low mitochondrial respiratory function in the skeletal muscle of patients with PD, which might be associated with motor impairment [20,73,74,75]. Clinical studies also observed a loss of efficiency in oxidative metabolism in the muscles of patients with PD [76,77]. Animal studies of PD-like conditions have also reported mitochondrial dysfunction in the skeletal muscles of rats treated with 6-OHDA [44] and Parkin-knockout mice [78]. Similarly, we observed unilateral 6-OHDA injection induced mitochondrial dysfunction in the skeletal muscles. However, HMP supplementation for 8 weeks restored the muscle mitochondrial function. The antioxidant effects of HMP supplementation were associated with higher expression of *Sod1* and the antioxidant transcriptional regulator *Nrf2* in muscles (Figure 3C) and higher total SOD activity in serum (Figure 4), and these changes may further reduce oxidative stress in muscles. The reduction in oxidative stress may lead to upregulation of mtDNA biogenesis (*Pgc1*) and transcription (*Tfam*), and result in the increased mtDNA copy numbers [complex 1 (*Nd1*) and complex V (*Atp6*)] (Figure 5C,D). Preservation of the mtDNA copy number may have improved mitochondrial function in skeletal muscles (Figure 5A,B) and thus prevented severe motor dysfunction (Figure 2A). MP treatment is known to increase the OCR [63] and the activities of oxidative respiratory enzymes, including nicotinamide adenine dinucleotide-cytochrome c reductase (NCCR), succinate-cytochrome c reductase (SCCR), and cytochrome c oxidase (CCO) [79]. Furthermore, MP supplementation could upregulate antioxidant enzymes and decrease mitoROS, which could prevent further mitochondrial damage [63].

Gut dysbiosis has been associated with the development of PD. Several studies have reported increased abundance of inflammation-related genera, such as *Streptococcus* [21], *Ralstonia* [22], *Akkermansia* [23], and *Sutterella* [24], and decreased abundance of anti-inflammation-related genera, such as *Prevotella* [25], *Blautia* [21], and *Roseburia* [22], in fecal samples from clinical and preclinical models of PD. Our previous study also demonstrated changes in the microbiota profile in a 6-OHDA-induced model of PD [40]. Herein, we observed similar alterations in the fecal microbiota profile. In the untreated PD group, the abundance of Proteobacteria at the phylum level and abundance of *Streptococcus* and *Sutterella* at the genus level were enriched, while *Lactobacillus* and *Roseburia* were reduced (Figure 8). HMP supplementation attenuated the alterations in these taxa induced by the 6-OHDA PD injection. MP contains beneficial polyphenols that may promote a gut environment that supports commensal bacteria and limits colonization of pathogenic microbiota in the gut [80]. Due to its capacity to modulate the gut microbiota profile, polyphenol-rich HMP may increase the antioxidant status (Figure 3 and Figure 4) and could prevent prolonged inflammation under PD-like conditions, as indicated by the observed decreases in the plasma levels of IL-1β, IL-6, and TNF-α (Table 3).

In most clinical studies, the abundance of *Prevotella* is reported to be lower in PD compared to healthy controls. However, Heintz-Buschart et al. found *Prevotella* sp. was more abundant in the gut of patients with PD than in healthy subjects [81]. *Prevotella* is associated with immunoregulatory function in the gut. Overgrowth of *Prevotella* in the gut is linked to mucosal inflammation through enhanced production of proinflammatory cytokines and chemokines, activation of T helper type 17 cells (Th17), and increased activation of neutrophils [26,82]. The genus *Rothia*, which is commonly found in the human mouth and upper respiratory tract, was reported to be highly enriched in the oral cavity of patients with PD [83]. A high abundance of *Rothia* is known to cause peritoneal infection [84] and cerebrospinal fluid infection [85] in immunocompromised patients. In an in vitro study, the species *Aggregatibacter actinomycetemcomitans* was shown to produce a leukotoxin that promoted the secretion of IL-1β from human macrophages [86]. In this study, HMP supplementation for 8 weeks suppressed the enrichment of *Rothia*, *Prevotella*, and *Aggregatibacter* in the PD rats (Figure 8). In addition, the increase in *Aggregatibacter* was accompanied by an increase in the level of IL-1β in plasma in the untreated PD group. Moreover, our correlation analysis suggested that the abundance of *Prevotella*, *Rothia*, and *Aggregatibacter* correlated negatively with antioxidant-related gene expression (Table 4). This suggests that HMP not only directly exerted antioxidant effects, but also indirectly increased antioxidant status through gut microbiota alterations. This finding further supports the suggestion that rebuilding of the gut microbiota composition may boost the antioxidant status of the host.

We also observed a trend towards increased abundance of the genus *Turicibacter* in groups supplemented with HMP, while the untreated PD group showed a slight decrease in this genus. This finding may be positively correlated with the observed improvements in antioxidant capacity at the systemic level and in the muscles and brain. *Turicibacter* is known to promote production of butyric acid in the gut [87] and upregulates antioxidant enzymes, such as SOD and GPx [27]. Furthermore, increased levels of butyric acid induced by *Turicibacter* may also possibly mediate the optimization of mitochondrial function observed in the muscles [88]. We also observed a positive correlation between *Turicibacter* and *Gpx* expression in the brain. These results further strengthen the evidence of a role for the gut microbiota in antioxidant defense mechanisms in PD-like conditions.

Here, we evaluated the effects of MP on DAT binding activity in our PD animal model using [^18^F]FE-PE21 PET scans. Although it may not represent the precise number of dopaminergic neurons in the brain, the [^18^F]FE-PE21 PET scanning technique used in this study is a reliable tool to estimate active dopaminergic neurons. Additionally, DAT expression can propose a potential surrogate marker for dopaminergic neurons in nigrostriatal pathway and can be used to predict motor function in PD [48]. Moreover, we propose that in addition to the indirect evidence of neuroinflammation, neuroinflammation in the brain could be directly assessed in future studies to characterize the evidence of neuronal damage of PD.

## 5. Conclusions

This study demonstrated that supplementation with MP extract, especially at the higher dose, delayed motor deficit in 6-OHDA-induced PD rats. The effects of MP may mainly be derived from direct and indirect activation of cascades in the cellular antioxidant defense system. The natural antioxidative compounds present in MP may directly increase endogenous antioxidants to counterbalance the excessive production of free radicals in the rat model of PD. MP may also indirectly boost antioxidant status by rebuilding the gut microbiota, which in turn reduces the levels of inflammatory cytokines, restores mitochondrial function, and ultimately prevents dopaminergic neuronal death and delays motor deficits. Additionally, this study provides insight into the importance of improving the antioxidant capacity and mitochondrial function of muscles to delay PD-like motor deficits. Overall, our study suggests the use of MP, specifically the higher dose, has the potential to support PD therapy.

## Figures and Tables

**Table 1:** Profiles of body weight (BW) gain, food intake, food conversion efficiency (FCE), and water intake after 8 weeks of supplementation in the rat model of PD.

Parameter	NC	PD	LMP	HMP
BW gain (g)/rat	246.4 ± 37.2	236.4 ± 18.0	224.0 ± 18.7	227.7 ± 10.7
Food intake (Kcal/day/rat)	107.3 ± 7.7	105.0 ± 4.7	96.9 ± 4.5	97.1 ± 2.5
FCE (%)	225.3 ± 20.9	227.8 ± 22.0	231.5 ± 15.9	238.2 ± 17.7
Water intake (mL)/day/rat	44.7 ± 2.1	43.1 ± 3.6	44.8 ± 1.3	44.7 ± 2.4

Data are average ± SEM for *n* = 5 rats/group. BW gain represents the increase in BW from the day the lesions were induced until the end of the 8-week supplementation. FCE represents BW gain/total calorie intake. No significant differences were found between groups in one-way ANOVA. NC, normal control; PD, untreated Parkinson’s disease (PD); LMP, PD rats supplemented with 30 mg/kg BW/day of mangosteen pericarp extract (MP); HMP, PD rats supplemented with 60 mg/kg BW/day of MP.

**Table 2:** Correlations between motor function and antioxidant parameters after 8 weeks of supplementation in the rat model of PD.

Motor Function Parameter	Correlation	Antioxidant Parameters
Time spent on rod (s)	+	Serum total SOD (*r* = 0.702; *p* < 0.0001), Brain *Sod1* (*r* = 0.470; *p* = 0.018), Brain *Cat* (*r* = 0.511; *p* = 0.009) Muscle *Sod1* (*r* = 0.610; *p* = 0.001), Muscle *Sod2* (*r* = 0.417; *p* = 0.020), Muscle *Cat* (*r* = 0.518; *p* = 0.008)
	−	Brain ROS level (*r* = −0.445; *p* = 0.026)

*Sod*, superoxide dismutase; *Cat*, catalase; ROS, reactive oxygen species; +, positive correlation; −, negative correlation. *p* < 0.05 was considered significant.

**Table 3:** Plasma levels of inflammatory cytokines after 8 weeks of supplementation in the rat model of PD.

Parameters	NC	PD	LMP	HMP
Plasma IL-1β (pg/mL)	382.5 ± 1.7	596.2 ± 6.7 ^a^	454.9 ± 2.5 ^b^	441.4 ± 3.0 ^b^
Plasma IL-6 (pg/mL)	100.9 ± 5.0	131.6 ± 4.3 ^a^	114.9 ± 6.2	108.2 ± 6.2 ^b^
Plasma TNF-α (pg/mL)	95.5 ± 2.1	130.9 ± 3.4 ^a^	112.6 ± 4.4 ^b^	101.9 ± 3.4 ^b^

Results are average ± SEM and were evaluated using one-way ANOVA with Tukey’s post hoc test (*n* = 5 rats/group). ^a^ *p* < 0.05 for the Parkinson’s disease (PD) group vs. the normal control (NC) group. ^b^ *p* < 0.05 for the low-dose MP (LMP) and high-dose MP (HMP) groups vs. the PD group. There were no significant differences between the LMP and HMP groups.

**Table 4:** Correlations of bacterial taxa with parameters of motor function, antioxidant and mitochondrial gene expression, and inflammatory cytokines after 8 weeks of supplementation in the rat model of PD.

Parameter		Correlation	Bacterial Taxa
Time spent on rod (s)		−	*Streptococcus* (*r* = −0.585; *p* = 0.002)
			*Rothia* (*r* = −0.663; *p* = 0.0003)
			*Rothia naismurium* (*r* = −0.663; *p* = 0.0003)
Brain ROS level (% normal control)		+	*Rothia* (r = 0.438; *p* = 0.028)
			*Rothia naismurium* (*r* = 0.438; *p* = 0.028)
Brain antioxidant-related genes (fold change)			
	*Sod1*	−	*Sutterella* (*r* = −0.459; *p =* 0.021)
			*Aggregatibacter* (*r* = −0.465; *p* = 0.019)
			*Rothia* (*r* = −0.412; *p* = 0.041)
			*Rothia naismurium* (*r* = −0.412; *p* = 0.041)
			*Aggregatibacter pneumotropica* (*r* = −0.465; *p* = 0.019)
	*Sod2*	+	*Rothia* (*r* = 0.473; *p* = 0.017)
			*Rothia naismurium* (*r* = 0.473; *p* = 0.017)
	*Cat*	−	*Sutterella* (*r* = −0.746; *p* < 0.0001)
			*Aggregatibacter* (*r* = −0.405; *p* = 0.045)
			*Streptococcus* (*r* = −0.428; *p* = 0.033)
			*Aggregatibacter pneumotropica* (*r* = −0.405; *p* = 0.045)
	*Gpx*	+	*Turicibacter* (*r* = 0.417; *p* = 0.038)
		−	*Prevotella* (*r* = −0.446; *p* = 0.025)
			*Sutterella* (*r* = −0.451; *p* = 0.024)
			*Streptococcus* (*r* = −0.302; *p* = 0.142)
	*Nrf2*	−	*Sutterella* (*r* = −0.404; *p* = 0.045)
Muscle antioxidant-related genes (fold change)			
	*Sod1*	−	*Lactococcus* (*r* = −504; *p* = 0.010)
			*Prevotella* (*r* = −0.417; *p* = 0.038)
			*Aggregatibacter* (*r* = −0.418; *p* = 0.037)
			*Streptococcus* (*r* = −0.463; *p* = 0.020)
			*Rothia* (*r* = −0.420; *p* = 0.037)
			*Rothia naismurium* (*r* = −0.420; *p* = 0.037)
			*Aggregatibacter pneumotropica* (*r* = −0.418; *p* = 0.037)
	*Cat*	−	*Lactococcus* (*r* = −0.411; *p* = 0.041)
			*Aggregatibacter* (*r* = −0.450; *p* = 0.024)
			*Streptococcus* (*r* = −0.455; *p* = 0.022)
			*Aggregatibacter pneumotropica* (*r* = −0.45; *p* = 0.024)
	*Nrf2*	−	*Sutterella* (*r* = −0.540; *p* = 0.005)
Serum total SOD (U/mL)		−	*Prevotella* (*r* = −0.409; *p* = 0.042)
	Muscle mitochondria-related genes (fold change)		
	*Nd1*	−	*Streptococcus* (*r* = −0.468; *p* = 0.038)
			*Rothia* (*r* = −0.453; *p* = 0.045)
			*Sutterella* (*r* = −0.447; *p* = 0.048)
			*Streptococcus* spp. (*r* = −0.468; *p* = 0.038)
			*Rothia nasimurium* (*r* = −0.453; *p* = 0.045)
	*Pgc1α*	−	*Streptococcus* (*r* = −0.560; *p* = 0.010)
			*Rothia* (*r* = −0.544; *p* = 0.013)
			*Streptococcus* spp. (*r* = −0.560; *p* = 0.010)
			*Rothia nasimurium* (*r* = −0.544; *p* = 0.013)
	*Nrf1*	+	*Coprococcus* (*r* = 0.455; *p* = 0.044)
		−	*Rothia* (*r* = −0.464; *p* = 0.039)
			*Rothia nasimurium* (*r* = −0.464; *p* = 0.039)
	*Tfam*	−	*Streptococcus* (*r* = −0.629; *p* = 0.003)
			*Rothia* (*r* = −0.636; *p* = 0.002)
			*Sutterella* (*r* = −0.516; *p* = 0.020)
			*Aggregatibacter* (*r* = −0.528; *p* = 0.017)
			*Streptococcus* spp. (*r* = −0.629; *p* = 0.003)
			*Rothia nasimurium* (*r* = −0.636; *p* = 0.002)
	Plasma inflammatory cytokines (pg/mL)		
	IL-1β	+	*Streptococcus* (*r* = 0.557; *p* = 0.011)
			*Rothia* (*r* = 0.708; *p* < 0.001)
			*Aggregatibacter* (*r* = 0.563; *p* = 0.010)
			*Streptococcus* spp. (*r* = 0.557; *p* = 0.011)
			*Rothia nasimurium* (*r* = 0.708; *p* < 0.001)
	IL-6	+	*Streptococcus* (*r* = 0.602; *p* = 0.005)
			*Rothia* (*r* = 0.649; *p =* 0.002)
			*Sutterella* (*r* = 0.585; *p* = 0.007)
			*Aggregatibacter* (*r* = 0.544; *p* = 0.013)
			*Streptococcus* spp. (*r* = 0.602; *p* = 0.005)
			*Rothia nasimurium* (*r* = 0.649; *p =* 0.002)
			*Coprobacillus* spp. (*r* = 0.580; *p* = 0.007)
	TNF-α	+	*Streptococcus* (*r* = 0.513; *p* = 0.021)
			*Rothia* (*r* = 0.583; *p =* 0.007)
			*Aggregatibacter* (*r* = 0.511; *p* = 0.021)
			*Streptococcus* spp. (*r* = 0.513; *p* = 0.021)
			*Rothia nasimurium* (*r* = 0.583; *p =* 0.007)
		−	*Lactobacillus* (*r* = −0.465; *p* = 0.039)

*Sod*, superoxide dismutase; *Cat*, catalase; *Gpx*, glutathione peroxidase; *Nrf2*, nuclear factor-erythroid factor 2-related factor 2; ROS, reactive oxygen species; *Nd1*, NADH dehydrogenase subunit 1; *Pgc1α*, peroxisome proliferator-activated receptor gamma coactivator 1-alpha; *Nrf1*, nuclear respiratory factor 1; *Tfam*, transcription factor A, mitochondrial; IL, interleukin; TNF-α, tumor necrosis factor alpha; +, positive correlations; −, negative correlations. *p* < 0.05 was considered significant.

## Data Availability

All data are contained within the article or Supplementary Materials.

## Supplementary Materials

The following supporting information can be downloaded at: https://www.mdpi.com/article/10.3390/antiox11122396/s1, Table S1: Primer sequences for rat antioxidant and mitochondrial mRNA; Figure S1: High-performance liquid chromatography (HPLC) elution profiles of mangosteen pericarp extract, γ-mangostin standard, and α-mangostin standard; Figure S2: Comparison of mitochondrial function and mitochondria-related mRNA expression in the brain between groups after 8 weeks of supplementation.
