# Supplementary material for: Mangosteen Pericarp Extract Supplementation Boosts Antioxidant Status via Rebuilding Gut Microbiota to Attenuate Motor Deficit in 6-OHDA-Induced Parkinson’s Disease
Source: Antioxidants (Basel). 2022 Dec 2;11(12):2396. doi: 10.3390/antiox11122396 (PMC9774421; doi:10.3390/antiox11122396)
Supplement: Supplementary file 1 [file antioxidants-11-02396-s001.zip › antioxidants-2007133-supplementary.pdf]

Supplementary Materials

Table S1. Primer sequences for rat antioxidant and mitochondrial mRNA

Gene	Forward	Reverse	Accession Number
Actb	5'- GCAGATGTGGATCAGCAAGC	5'- GGTGTAAAACGCAGCTCAGTAA	NM_031144.3
Sod1	5'- TCTAAGAAACATGGCGGTCC	5'- CAGTTAGCAGGCCAGCAGAT	NM_017050.1
Sod2	5'-CAATCTGAACGTCACCGAGGA	5'-AGCAGGCGGCAATCTGTAA	NM_017051.2
Cat	5'-TGTGGTTTTCACCGACGAGAT	5'-GCTTGAAGGTGTGTGAGCCAT	NM_012520.2
Gpx	5'- CTCTCCGCGGTGGCACAGT	5'- CCACCACCGGGTCGGACATAC	NM_030826.4
Nrf2	5'- TCAATGACTCTGACTCCGGCA	5'- TCCTACAGTTCTGAGCGGCAA	NM_001399173.1
Nd1	5'- CAAAGGCCCCAACAACGAAG	5'- TGAGGTGGTTAGAGGGCGTA	X07479.1
Atp6	5'- CGAACCTGAGCCCTAATA	5'- GTAGCTCCTCCGATTAGA	MW209726.1
Pgc1a	5'-GTGCAGCCAAGACTCTGTATGG	5'-GTCCAGGTCATTCACATCAAGTTC	NM_031347.1
Nrf1	5'- GCTGTCCCACTCGTGTCGTAT	5'- GTTTGAGTCTAACCCATCTATCCG	NM_001100708.1
Tfam	5'- CGCCTAAAGAAGAAAGCACA	5'- GCCCAACTTCAGCCATTT	NM_031326.2

(A)

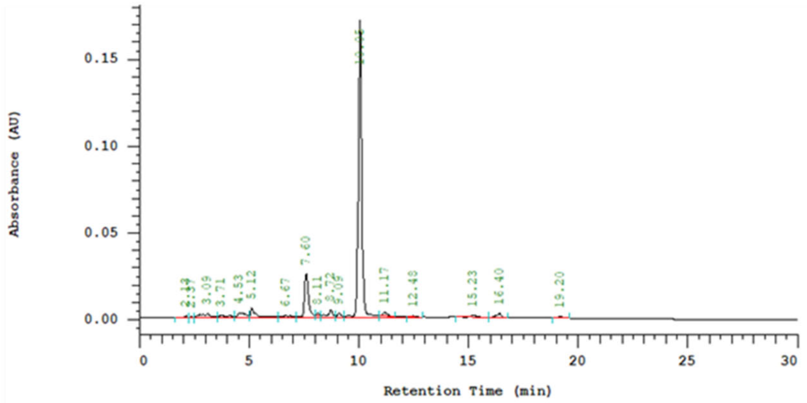

(B)

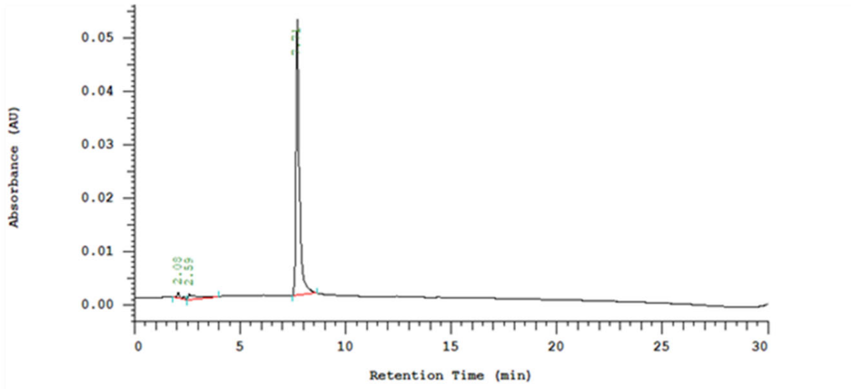

(C)

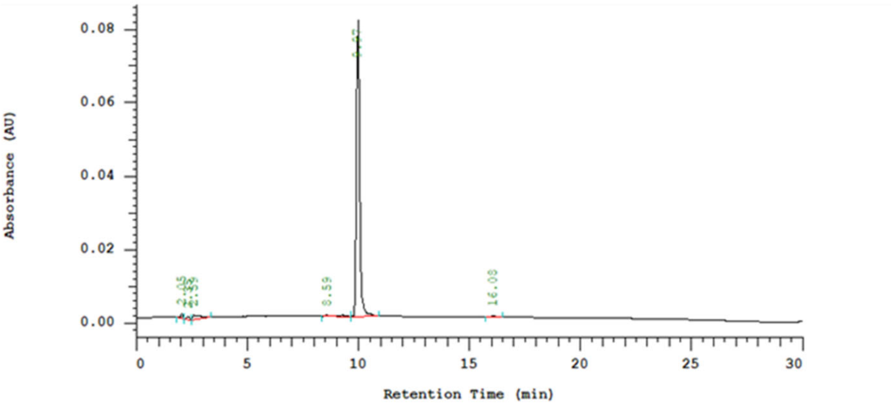

Figure S1. High-performance liquid chromatography (HPLC) elution profiles of (A) mangosteen pericarp extract (0.1 mg of mangosteen pericarp extract/mL), (B) γ-mangostin standard (25 µg/mL), and (C) α-mangostin standard (25 µg/mL).

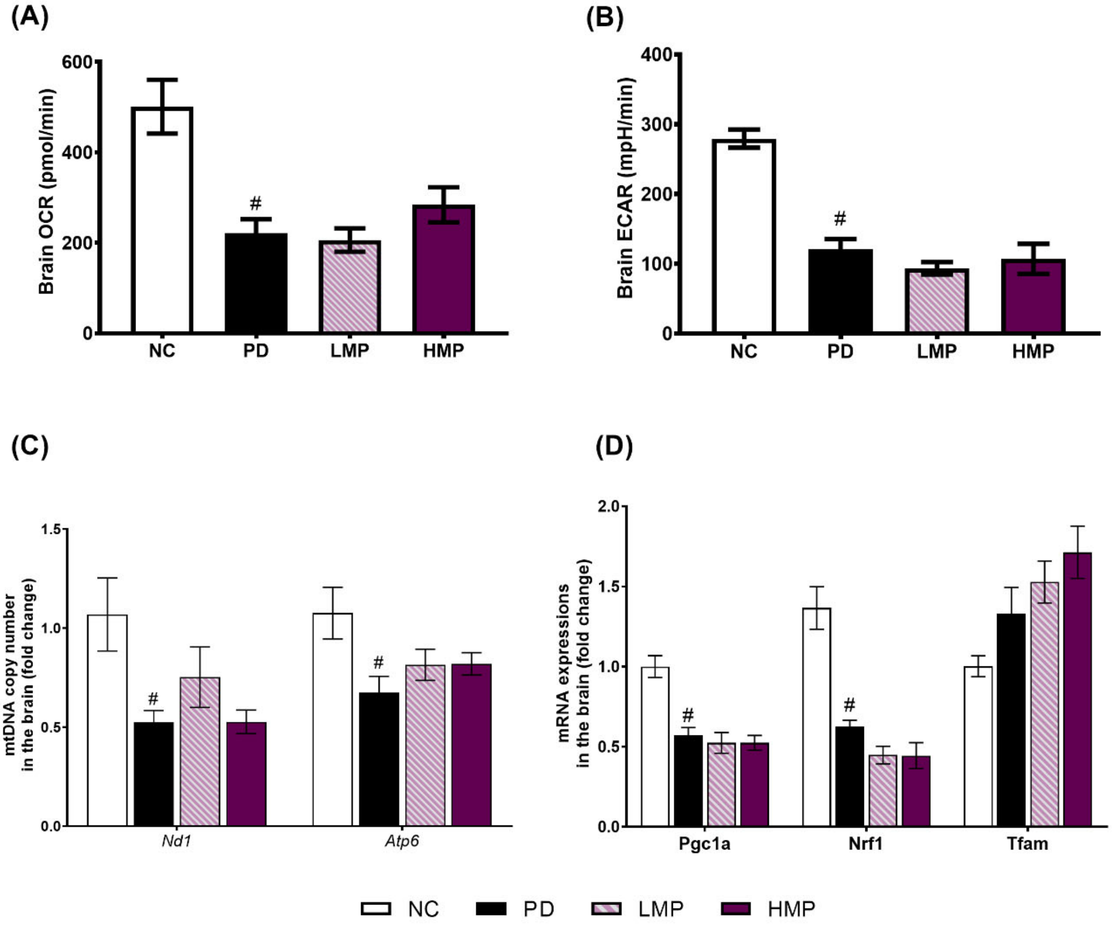

Figure S2. Comparison of mitochondrial function and mitochondria-related mRNA expressions in the brain between groups after 8-weeks supplementations. **(A)** The basal oxygen consumption rate (OCR) as used to evaluate mitochondrial respiration. **(B)** The basal extracellular acidification flux (ECAR) was used to evaluate the glycolysis rate. **(C)** mtDNA copy numbers (*Nd1* and *Atp6*). **(D)** Mitochondria biogenesis-related mRNA expression genes: PPARG coactivator 1 alpha (*Pgc1a*), nuclear respiratory factor 1 (*Nrf1*), mitochondrial transcription factor A (*Tfam*). Results are presented as the average ± SEM with n = 5 rats/group as analyzed by one-way ANOVA with Tukey's post hoc test. $^{\#}$ $p < 0.05$ in a comparison of the Parkinson's disease (PD) group with the normal control (NC) group. No significant differences found between PD group and low-dose mangosteen pericarp (LMP) or high-dose mangosteen pericarp (HMP) group.
